# Optically active spins in van der Waals materials and devices

**DOI:** 10.1557/s43577-026-01062-6

**Published:** 2026-03-19

**Authors:** Carmem M. Gilardoni, Hannah L. Stern, Mete Atatüre

**Affiliations:** 1 Centro Brasileiro de Pesquisas Físicas, Rio de Janeiro, 22290-180 Brazil; 2 Department of Materials, University of Oxford, Oxford, OX1 3PH UK; 3 Cavendish Laboratory, University of Cambridge, Cambridge, CB3 0US UK

**Keywords:** Defects, Devices, Heterostructure, Layered, Photonic, Single-photon source/emitter, Spectroscopy, Spin, van der Waals

## Abstract

**Abstract:**

Layered materials offer a singular, versatile platform for the development of quantum communication and sensing applications based on optically addressable spins. Insulating and semiconducting layered materials host optically addressable spins that can be created via top-down and bottom-up approaches, and recent advances with photonic and electronic devices can achieve *in situ* manipulation of their optical and spin transitions. Combined with the large variety of naturally occurring and artificially synthesized layered materials, van der Waals (vdW) materials provide extensive opportunities, from novel defect engineering to scalable device engineering. However, challenges include identification of the microscopic configuration of the atomic and electronic structures that give rise to optically addressable spins in these materials, as well as achieving the desired level of reproducibility at defect, material, and device levels simultaneously. Here, we present an overview of the recent advances in these areas, including a discussion of the microscopic origin of some of the quantum emitters in vdW materials, as well as strategies toward developing functional devices based on these systems.

**Graphic abstract:**

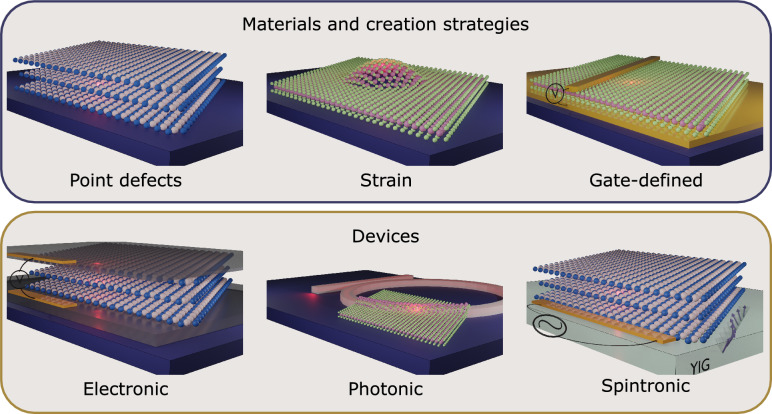

## Introduction

Communications and sensing strands of emerging quantum technologies require integrating qubits that can interface photonic and stationary degrees of freedom into operational devices. Material-based qubits that offer optically addressable spins enable distant spins to interact with each other via long-range exchange of flying qubits,^1–4^ lifting the requirement of proximity for entanglement. In this way, optically addressable spins that offer a coherent spin–photon interface (SPI) are a clear route to a distributed technology, and may be the critical solution to interconnection of even densely packed systems. Their technological implementation relies on integrating these systems into electronic,^5,6^ photonic,^4,7,8^ spintronic,^9^ and mechanical^10^ devices that are a resource for creating,^11,12^ stabilizing,^13^ tuning,^6,7^ and controlling^14^ their active quantum states. For example, electronic devices enable on-demand generation of single photons through electrical, rather than optical, excitation;^5^ photonic devices have an important role in enhancing photon emission and photon collection from solid-state single-photon emitters;^15^ spintronic devices may facilitate control and readout of spin degrees of freedom,^9,14^ enhancing the fidelity of gate operations of spin-based systems for quantum sensing, computing, and communication. Finally, mechanical devices may provide a pathway for integrating optically active quantum systems to optically inert systems such as superconducting qubit platforms.^10,16^

The development of such devices requires the combination of functional properties provided by different materials: metallic, insulating, and semiconducting materials for electronic devices, high-dielectric materials for photonic devices, magnetic materials for spintronic devices, and piezoactive or flexible materials for mechanical devices. In this context, van der Waals (vdW) materials present unique compatibility and ease of integration into various device architectures, largely due to their layered character, the variety of functional materials available in vdW form, and their large degree of tunability.^17,18^ This motivates the search for optically addressable spins in vdW materials and their heterostructures, and the investigation of these systems when integrated into functional device architectures. In particular, optically addressable spins in vdW materials that function as quantum sensors in vdW material platforms may provide significant improvements on the maximum sensitivity achieved when compared to the more conventional bulk-semiconductor platforms, as the dimensionality of the material facilitates photon extraction and enables operation at smaller sensor-target distances.^19–21^ This article provides an overview of the most widely studied optically addressable spins in vdW materials, the proposed origins of the localized quantum states, and the current progress and challenges involved in integrating these systems into active devices.

## Optically addressable spins in van der Waals materials

A unifying property of the plethora of vdW materials is that they are layered anisotropic crystals with covalent or ionic bonds within layers and weak vdW bonds between adjacent layers. This chemical configuration means some vdW materials can be obtained as a single (monolayer [ML]) or a few constituent layers through layer-by-layer chemical growth or mechanical exfoliation from bulk crystals. For integration into functional devices, the versatility of vdW materials arises from the fact that ML and few-layer-thick vdW materials can be combined via stacking techniques and placed on different substrates forming atomically flat interfaces, irrespective of matching between lattice parameters or crystalline orientations.^17^ This is in contrast to the technically more demanding epitaxial growth techniques required to combine different bulk materials,^22,23^ and gives rise to a unique degree of freedom when combining different vdW materials together: the constituent layers can be rotated with respect to each other to create superlattices and modify the electronic states.^24–26^ Further, proximity effects can be used to tune electronic properties when materials of different character are combined.^27,28^ Finally, ML materials hosting optically addressable spins with quantum sensing capabilities offer significant improvement on the sample-target distance of operation when compared to what is currently achieved with bulk-semiconductor sensors.

### Transition-metal dichalcogenides

Transition-metal dichalcogenides (TMDs) constitute a semiconducting class of vdW materials with bandgap energy typically between $\sim$1.3 and $\sim$1.6 eV, including but not limited to MoS$_2$, MoSe$_2$, WS$_2$, WSe$_2$.^29–31^ In the unperturbed case, the electronic states at the edges of valence and conduction bands of MLs present coupled spin, valley and layer-number degrees of freedom with strong optical selection rules and highly efficient exciton emission, owing to the combination between spatial symmetries of the material and spin–orbit coupling.^32^

**Figure 1:**
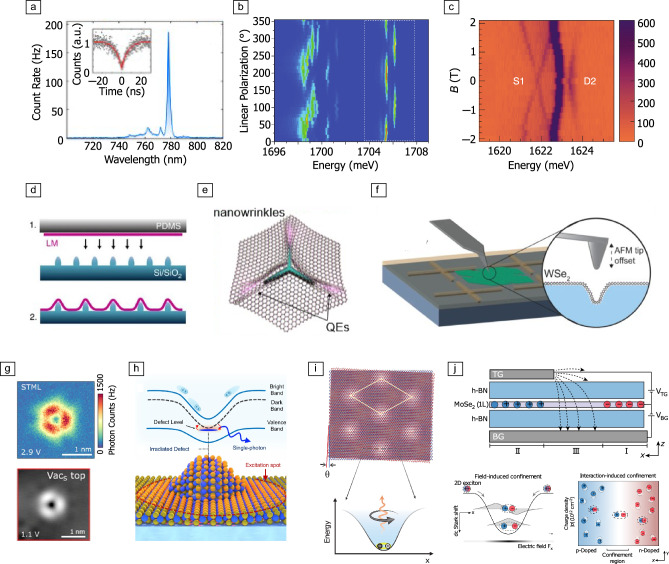
Localized emitters with spin interfaces in transition-metal dichalcogenides (TMDs). (a) Optical emission spectrum of a localized emitter created via strain profile in WSe$_2$, with inset showing second-order photon-correlation that is evidence of the presence of a single-photon emitter.^56^ (b) Photoluminescence of a localized emitter in WSe$_2$ as a function of optical polarization of emitted photons, showing signatures of cross-linearly polarized doublets.^46^ (c) Photoluminescence from two different emitters in WSe$_2$, labeled as S1 and D2, as a function of applied magnetic field. Emitter S1 has degenerate emission features at zero magnetic field that evolve linearly, and is assigned to a positively charged localized trion emitter. Emitter D2 presents a doublet at zero magnetic field with an anticrossing feature as the absolute magnetic field increases, and is assigned to a localized neutral exciton emission.^42^ (d–f) Mechanisms for the deterministic creation of localized emission sites in monolayer (ML) TMDs, involving the transfer of TMD MLs onto (d) nanostructured substrates.^11^ LM, layered material; PDMS, poly(dimethylsiloxane). (e) Nanowrinkles on hBN^57^ and (f) the indentation of ML TMDs using scanning probe tips.^35^ QEs, quantum emitters; AFM, atomic force microscope. (g) Scanning tunneling microscope luminescence (STML) (top) scan over a chalcogen vacancy in WS$_2$ (top), correlated to a scanning tunneling microscopy (STM) topography map of the same chalcogen vacancy.^33^ (h) Schematic representation of a model that proposes that strain gradients give rise to localized photon emission in ML TMDs due to hybridization between dark excitonic states and defect states.^64^ (i) Schematic representation of Moiré patterns created via the combination of TMD MLs rotated with respect to each other. The spatial variation of atomic configuration within the artificial bilayer generates a potential well for excitons that can give rise to single-photon emission.^38^ (j) A depiction of a top-down device that gives rise to exciton localization via external applied gates.^36^ TG, top gate; BG, bottom gate.

Due to the relatively low bandgap of the materials, localized quantum light emission typically arises from the excitonic states of the unperturbed materials locally modified by electrostatic traps arising from the presence of shallow atomic defects^33^ and/or the application of strain,^11,34,35^ electric fields,^36^ and periodic potentials.^37,38^ This leads to quantum-dot-like systems hosting excitonic states that inherit the spin-photon coupling properties of the host materials.^39–44^ An example of a photoluminescence spectrum, with below-bandgap quantum light emission, is shown in **Figure** **1**a. The low trapping energies, when compared to those provided by atomic lattice defects, result in quantum emission that is typically restricted to cryogenic operation.^45^

Different types of single-photon emitters can emerge in ML TMDs: on the one hand, emitters with doublet emission signatures at zero magnetic field, observed predominantly in WSe$_2$ and WS$_2$, have cross-linearly polarized emission at zero magnetic field that evolve to cross-circularly polarized optical features at high magnetic field^39,46,47^ (see Figure 1b). This is a signature of coupling between spin, valley, and optical degrees of freedom. For these emitters, magneto-photoluminescence studies reveal a g-factor of $\sim$8,^42,44,46,48,49^ twice that of the bright free exciton,^50^ supporting the hypothesis that local brightening of dark neutral exciton species mediated by strain or hybridization with local defect states is responsible for these single-photon emitters.^51^ Polarization-dependence of these spin-resolved emission features indicate fast relaxation of the valley degree of freedom, a challenge that must be overcome for wide implementation of these emitters as SPIs.^42,51^ On the other hand, localized positively charged trion emission results in degenerate optical transitions at zero magnetic field that evolve linearly to two emission features under applied field with a Zeeman splitting governed by a g-factor close to $\sim$12.^42^ Upon recombination of the electron–hole pair, the additional hole of the trion is initialized into a well-defined spin-polarized state,^42^ indicating these systems could function as optically addressable solid-state quantum memories. An example of the Zeeman-behavior of doublet and singlet features is presented in Figure 1c.^42^

Initial investigations of single-photon emitters in ML TMDs relied on the stochastic creation of quantum-dot-like features by uncontrolled strain.^39–41,43,44,46,47,52^ However, the search for deterministic creation of quantum emitters with controlled photon-emission properties has led to significant advances in the last 10 years, where different experimental approaches have been explored. Quantum emitters in the red and near-infrared ranges of the optical spectrum have been created in ML WSe$_2$ and WS$_2$ by harnessing strain gradients that appear when the materials are transfered onto nanopatterned dielectric substrates (Figure 1d),^11,34,53,54^ glassy substrates with irregular surface profiles,^55^ and wrinkled hBN (Figure 1e),^56,57^ or subject to nanoindentations due to force applied with atomic force microscopy tips (Figure 1f).^35^ Further, the creation of hydrogen-filled nanodomes in the material through irradiation has also been a successful strategy for generating localized quantum emitters.^49^ For MoS$_2$, strain profiles alone give rise to broad, defect-related excitonic emission bands,^58^ but the successful path for deterministic creation of single-photon emitters has so far required irradiation of the material with ion or electron beams,^59,60^ leading to chalcogen vacancies.

The apparent discrepancy between creation strategies of similar quantum emitters in the various TMDs leads to continued discussion on their microscopic origin.^61,62^ Despite deterministic strain patterns being an effective way to create quantum emitters in WSe$_2$ and WS$_2$,^34,54,56^ scanning tunneling microscope luminescence studies of ML WS$_2$ have revealed that, similar to what is seen in the case of Mo-based TMDs, chalcogen-vacancy states play a prevalent role in generating localized exciton emission in this material (Figure 1g).^33^ Deconvoluting the individual roles of strain and defect states in these quantum emitters has been a longstanding challenge^35,61^ where difficulty has risen from the fact that the influence of various different degrees of freedom—strain, defect states, dielectric interactions with the substrate and surrounding materials—further hinders the comparison between experimental results and first-principle predictions. Recently, theoretical^63^ and experimental^64,65^ works alike indicate that the most likely origin of quantum emitters in two-dimensional (2D) semiconductors is hybridization between defect and dark-exciton states, brought into resonance due to local strain-induced variations of the excitonic energy, as depicted schematically in Figure 1h.

In recent years, new approaches for creating traps for excitonic species in TMDs leading to spatially resolved single-photon emission have been explored. On the one hand, the Moiré superlattices arising from bilayers combining TMD MLs with different lattice parameters or crystalline orientations^24,25^ give rise to periodic modulations of the excitonic potential in these heterostructures.^66,67^ As depicted schematically in Figure 1i, when the period of modulation approaches the exciton radius, single excitons can be trapped within the potential wells, leading to theoretical predictions^37^ and experimental demonstrations of quantum light emission.^38,68,69^ The Moiré potential naturally gives rise to periodic arrays of quantum wells,^70^ where the barrier for tunneling between wells can be controlled externally through applied electric field.^71^ Additionally, these Moiré heterostructures present additional versatility arising from the variety of possible materials available and relative orientations between the MLs. Combining materials with different bandgaps^72^ or applying an out-of-plane electric field further enables control of the localization of charge carriers in different layers,^73–76^ giving rise to interlayer excitons that have optical lifetime far exceeding those of ML excitons and interact strongly with out-of-plane electric fields.^77^ Combined, these features place Moiré heterostructures based on TMDs as interesting platforms for exploring quantum simulation based on bosonic interactions.^78–80^

In parallel, fully electrostatic-gate-defined devices where excitons in ML TMDs are trapped locally due to electric-field gradients have been explored in the past three years. Typically, excitons tend to dissociate in the presence of in-plane electric field,^81^ a challenge that has previously hindered the investigation of excitons in gate-defined structures. However, recent advances in the design of gate architectures (Figure 1j), combined with the large binding energy and anisotropic behavior of excitons in ML TMDs have enabled the observation of localized light emission from excitonic quantum dots that are electrostatically defined.^12,36,82^ These systems, at their infancy, may provide top-down mechanisms to overcome challenges related to the inherent inhomogeneity of quantum emitters created through strain or TMD stacking.

### Hexagonal boron nitride

Hexagonal boron nitride is wide-bandgap (6 eV) vdW crystal that has been experimentally and theoretically shown to host a range of atomic-point defects in both monolayer and multilayer material.^8,83–87^ Unlike single-photon emitters in TMDs, emission in hBN is generally regarded to arise from strongly localized electronic states that sit energetically within the wide bandgap. Since the first identification of single photon emitting defects in hBN in 2016, several atomic defect types have been observed, the majority of studies focusing on multilayer hBN.^83,85,88–93^

**Figure 2:**
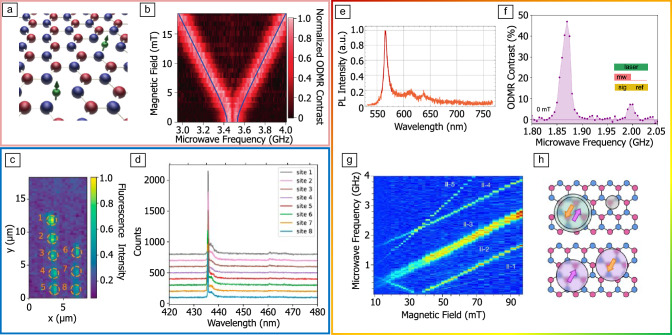
Optically active defects in hexagonal boron nitride (hBN). (a) Representation of the V_B_^-^, the most established defect center in hBN,^85^ and (b) optically detected magnetic resonance (ODMR) spectrum of an ensemble of V_B_^-^ as a function of applied magnetic field. (c) Photoluminescence spatial map of an irradiated multilayer hBN sample, showing localized single-photon emission features with (d) reproducible optical emission spectra at 4 K.^100^ (e) Spectrally resolved photoluminescence (PL) of a single emitter in the green-red range of the optical spectrum, with representative zero-phonon line and phonon sideband emission.^160^ (f) ODMR spectrum of a single defect optically active in the green-red range of the optical spectrum at ambient conditions, with signatures of a spin-1 defect and spin contrast reaching 50 percent.^92^ (g) ODMR map of a single defect in irradiated hBN, with signatures of both a >GHz zero-field-splitting (ZFS) spin-1 manifold, and sub-100 MHz ZFS spin-1 manifold.^112^ (h) Representation of an optical spin defect pair model proposed to explain the coexistence of the spin features presented in (g), consisting of two neighboring defects subject to charge-transfer, where co-localized charges give rise to a large ZFS, whereas charges distributed between the two defect sites give rise to a low ZFS.^118^

The most well-studied defect in hBN is the negatively charged boron vacancy (V$_{\text {B}}^{-}$, **Figure** **2**a), which can be formed readily via neutron, ion, or laser-beam irradiation. Due to its weak radiative transition, the V$_{\text {B}}^{-}$ is typically only detectable on the ensemble level and therefore is not a good single-photon emitter. However, due to optically detected magnetic resonance (ODMR, Figure 2b) and electronic paramagnetic resonance (EPR) studies combined with density functional theory (DFT), its electronic structure is now well understood.^85,86,94^ The V$_{\text {B}}^{-}$ has a spin-1 ground state with a zero-field splitting of $\sim$3.5 GHz, which is coupled via spin-dependent intersystem crossing to a spin-0 metastable state, in a similar way to the negatively charged nitrogen-vacancy (NV) center.^86^ Due to the out-of-plane spin quantization axis, the ability to layer hBN crystals containing high concentrations of V$_{\text {B}}^{-}$ with other materials, as well as the sensitivity of hBN lattice parameter to external factors, several recent studies have shown the V$_\text {B}^{-}$ is a promising quantum sensor of magnetic field,^20,95–97^ temperature,^95^ and pressure.^95,97^

Single defects that emit in the UV and blue regions of the optical spectrum have been deterministically created through carbon-doping^98^ and electron-beam (e-beam) irradiation of hBN,^99,100^ respectively (see Figure 2c). As illustrated in Figure 2d, these emissive features are spectrally narrow at 4 K and highly stable, with well-defined zero phonon line frequency (302 nm and 437 nm, respectively).^100–104^ Like the majority of emissive hBN defects known to date, the UV and blue emitters are associated with carbon impurities—UV emitters are assigned to a carbon dimer^98,105^ whereas blue emitters are currently thought to arise form carbon tetramers.^106–108^ Despite their promising optical properties, these defects lack any observable spin resonance to date.

In contrast, isolated single defects with optically addressable spins have been observed emitting in the the green-red region of the optical spectrum,^83,90–92,109–112^ with an example photoluminescence spectrum shown in Figure 2e. To date, hBN defects of this type display a range of magneto-optical properties that make them exciting candidates for quantum memory and sensing applications.^87^ These properties include the observation of room-temperature ODMR signatures on the single defect level (Figure 2f–g)—a demonstration that the electronic spin can be addressed independently via microwaves—high ODMR contrast ranging up to 100%,^92,112^ electronic spin coherence times on the $\upmu$s time scale,^113^ and coherent coupling to nuclear spins.^112^

Spin-active hBN defects that emit in the green-red spectral region fall into several classes. The first group show ODMR signatures that reveal an electronic spin-1 manifold with $\sim$ 2 Hz zero-field splitting and room-temperature spin coherence,^92,112,114^ akin to the widely studied NV center in diamond. For these electronic spins, the spin coherence ($T_{2}^\text {echo}$) is $\sim$200 ns and can be extended via dynamical decoupling to $\upmu$s time scales.^92^

Other single hBN defects present “spin-1/2” like ODMR resonances, arising from an electronic spin-1/2 or a spin-1 with weakly interacting individual electronic spin dipoles resulting in a sub-100 MHz zero-field splitting.^90,91,93,115,116^ As shown in Figure 2g, in some cases, “spin-1/2” like and spin-1 signatures have been observed simulataneously for single defects,^92,93,112,117^ a feature that is inconsistent with traditional point-like defects. This observation has motivated an intriguing model called the optical-spin defect pair (OSDP) that invokes charge transfer between pairs of optically active and inactive defects in the lattice,^117–120^ presented schematically in Figure 2h. This model, largely inspired by the behavior of molecular donor–acceptor or radical pairs^121^ provides a mechanism for optical initialization of “spin-1/2-like” defects, the coexistance of spin-1 and spin-1/2 signatures in some defects, and the overall variability of spin-related behavior observed for defects in hBN. Interestingly, studies with isotopically enriched samples have shown that the spin-1/2 ODMR resonances for defects of this type show clear hyperfine coupling to the $^{13}$C nuclear spin and this has been used to demonstrate coherent electro-nuclear coupling, a significant demonstration on the path toward establishing a nuclear-spin memory with a photonic interface based on hBN.^112^

The range of different spin defects emerging in hBN with similar optical properties highlights the difficulty in determining the microscopic configurations,^122^ an aspect that hinders the reproducibility of important experimental demonstrations and challenges the design of deterministic approaches to fabricating and stabilizing specific defects in the material.^122,123^ The similarity between the crystalline structures of hBN and graphene, and the flexibility of $sp^{2}$ hybridized orbitals forming the in-plane chemical bonds, leads to a particular versatility of hBN as host of molecular-like defects with complex chemical structures.^106,124,125^ Tens of different optically active carbon-related defects have been theoretically predicted to be stable in hBN and identified in scanning tunneling microscope studies.^124–126^ Further, theoretically predicted defect configurations may include interlayer bonding through impurities^107^ and out-of-plane deformations^127,128^ that stabilize otherwise unstable chemical structures. This leads to a particular difficulty in narrowing down defect configurations responsible for experimentally observed optical and spin properties.

Deterministic creation of specific optical defects is also a challenge. To date, creation of optically active defects has been demonstrated using various techniques, including irradiation with electron,^129^ helium,^130^ oxygen,^131^ and CO$_2$ beams,^112,132^ exposure to oxygen plasma^133^ annealing in an oxygen atmosphere,^134^ epitaxial growth of hBN in the presence of carbon precursors^108,122,135^ and over carbon-doped substrates,^123^ and nanopatterning the strain profile of hBN.^136–138^ Although defect centers created using these strategies vary significantly in their photophysical properties and spin character, they share similar optical emission spectra that include pronounced zero-phonon emission lines and a phonon sideband with strong signatures of coupling to phonons of the hBN lattice.^83,91,92,139,140^

## Device engineering

### Electronic devices

Both passive devices—consisting of combination of materials with different dielectric properties—and active devices—where external biases are used to manipulate the Fermi level and electric field in the device—have been shown to provide access to different degrees of freedom of optically addressable spins in vdW materials, where they’ve been useful for stabilizing, tuning, and controlling their quantum properties.

**Figure 3:**
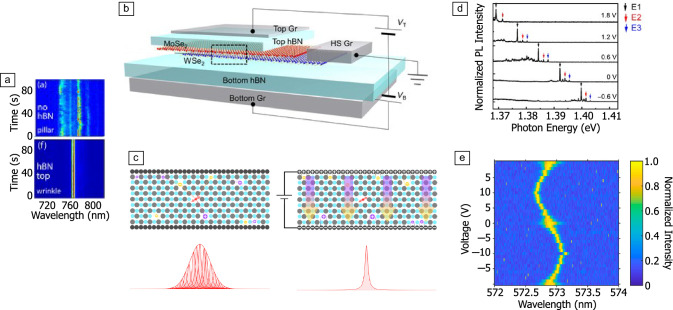
Electronic devices containing spin–photon interfaces in transition-metal dichalcogenides (TMD)s and hBN. (a) Photoluminescence (PL) spectrum of an emitter in exfoliated ML WSe$_2$ on a SiO$_2$ nanopillar over the course of 90 s (top), and for an emitter in exfoliated monolayer (ML) WSe$_2$ on an hBN nanowrinkle (bottom), with noticeable difference in spectral stability between the two.^56^ (b) Schematic of a typical architecture used for controlling the Fermi level and electric field in TMD devices, consisting of the TMD material (in this case a heterobilayer of MoSe$_2$/WSe$_2$) contacted independently through a graphene contact, with independent top and bottom graphene gates. Thin few-layers hBN are used as a dielectric. (c) Schematic of a device consisting of hBN with emissive defects sandwiched between two graphene gates, without (left) and with (right) applied bias between the electrodes. An electric field between the two gate electrodes leads to suppression of charge dynamics in the hBN few-layer structure, resulting in defect emission features with reduced inhomogeneous broadening (bottom schematics). (d) PL spectra from the device represented in (b), with peaks arising from emission at localized Moiré sites, as a function of electric field.^68^ (e) PL excitation spectra of an emissive defect in the device represented in (c) as a function of the bias between the two graphene electrodes, where both Stark tuning and a reduction of the emission linewidth under applied electric field are evident.^142^

Progress of technlogical applications based on optically addressable spins in vdW materials has been hindered by challenges in stabilizing the optical transition, which typically suffers from spectral wandering^141^ and blinking due to dynamically evolving charges within the material or in the environment,^39,41,56,57,142^ as illustrated in **Figure** **3**a. This effect is prevalent across different classes of optically addressable spins in both hBN and TMDs, and is also a well-known challenge for shallow defects in bulk crystals such as diamond.^143^ Mitigation strategies include encapsulating the active vdW material in an insulating material (hBN) in order to increase the distance to surface charges,^56,144^ and placing metallic layers,^144,145^ for example, graphene or gold, capable of screening charge-noise effects between the active material-substrate and active material–air interfaces. Further, strain profiles created using wrinkled hBN strucutures, rather than lithographically created pillars or domes, provide cleaner interfaces that significantly reduce these spurious effects, as illustrated in Figure 3a.^56,57^ These passive electrostatic device architectures have proven successful in significantly reducing the effects of spectral wandering for TMD-based spins,^56,144^ and inhomogeneous broadening between different defects for hBN-based devices.^145,146^

Overcoming detrimental charge state dynamics is critical for realizing lifetime-limited optical linewidths. One route is via application of an electric field to deplete shallow donor and acceptor states whose dynamics contribute to spectral wandering and blinking.^142,145,146^ Vertical devices where the active material is sandwiched between two gates (and hBN dielectric layers, in the case of TMDs) capable of generating a strong out-of-plane electric field, illustrated schematically in Figure 3b–c, have been used to demonstrate up to 1000-fold reduction of the optical linewidth of green-red quantum emitters in hBN at cryogenic temperature.^142,146^ In these works, the optical linewith is of the order of 100 MHz, nearly reaching the lifetime limit of $\sim$43 MHz. For emitters in WSe$_2$, the charge depletion and applied field across the device leads to a significant suppression of nonradiative decay pathways of excitons, resulting in increased brightness and near-unity quantum efficiency,^144^ although with optical linewidths still orders of magnitude above the lifetime limit. In bulk semiconducting materials, suppression of charge noise in engineered electronic devices of this type also contributes an increase in spin coherence due to suppression of paramagnetic noise,^13^ a feature that has not been demonstrated yet in layered-material-based devices.

The device architecture previously mentioned enables both charge control of quantum emitters due to Fermi-level control and tuning of the optical emission frequency via the Stark effect. In ML TMDs, the charge state of localized exciton emission can be controlled via independent top and bottom gates,^39,147,148^ with the previously mentioned spin-active doublet-emission features being activated at specific configurations.^39,42^ Control of the localized emission energy in these vertical devices is often hindered by the fact that excitons in ML TMDs have an in-plane dipole which is insensitive to out-of-plane electric field.^51^ In contrast, localized interlayer excitons in Moiré heterostructures have a pronounced out-of-plane electric dipole, which has enabled tuning of the optical transition by approximately 40 meV,^68^ $\sim$500 times the 80 $\upmu$eV optical linewidth of the localized exciton emission (Figure 3d). This impressive range stems partially from the device architecture, made possible through integration of different vdW materials – the use of thin vdW-material-based dielectric layers (e.g., hBN) allows for high electric field at modest voltages. In comparison, devices based on traditional bulk semiconductors such as SiC or diamond would require an applied voltage more than 10 times larger in order to achieve the same order of magnitude electric field. This is an important feature for bringing various inhomogeneously broadened emitters into resonance with each other.

This dual independent operation is easier to implement in TMD-based devices, where the chemical potential of the active TMD material can be uniformly addressed with respect to either graphene electrode.^77^ In contrast, in the case of hBN as an active SPI host, the large bandgap of the material prevents effective gate control of the chemical potential within the hBN, leading to experimental results where charge-state and electric-field control are intertwined.^142,149,150^ Despite this, gate-bias-induced brightening of optical signatures of individual emitters has been demonstrated.^146,151^ For defects emitting in the green-red region of the spectrum, independent results have demonstrated a tunability of the optical transition energy of 400 GHz under out-of-plane electric field at cryogenic temperature (Figure 3e),^142^ and >7 THz under in-plane electric field at room temperature.^152^

Finally, the ultimate goal for scaling up electronic devices hosting quantum-light emitters and spin–photon interfaces is to establish efficient electroluminescence and readout of spin states through electronic signals. In this respect, single-photon emission upon electronic excitation has been demonstrated for both TMD SPEs^51,52,115^ and defects in hBN,^109,149^ albeit with modest single-photon purity due to large background luminescence from either free excitonic emission in TMDs, or spurious defects in hBN. To the best of our knowledge, electrical readout of spin states has not been demonstrated in vdW materials yet. In bulk material platforms hosting SPIs, photon-mediated spin-to-charge conversion has enabled readout of spin coherence with 80% single-shot readout fidelity.^153^ In this case, the design of control protocols, including optical, gate voltage and microwave spin control pulses required detailed understanding of the defect’s photophysical and charge cycles. This knowledge is still being mapped out for most defects in hBN and TMDs. Nonetheless, defects whose spin properties stem from donor–acceptor pairs in hBN^119,120,154^ hold extensive potential for the independent and intricate control of the charge configuration of individual spins within defect configurations, opening various avenues for the possibility of establishing spin-to-charge conversion protocols for these defect types.

### Photonic devices

Integration of SPEs and SPIs into photonic devices, for example, photonic cavities and waveguides, and plasmonic structures, contributes to enhanced light–matter coupling that improves key figures of merit for the development of photonic quantum technologies, such as photon emission and collection rates^15^ and spectral stability.^7^ Additionally, photonic devices favor directional collection and propagation of light emitted by quantum light sources, being a fundamental step toward on-chip integration into multicomponent photonic circuit architectures.^155^

SPIs in vdW material hosts are particularly sensitive to photonic interactions with the environment, as demonstrated by recent results showing that the optical response of quantum emitters in hBN is strongly influenced by defect depth in a nontrivial manner due to a combination between plasmonic enhancement arising from interaction with the substrate and interference effects for excitation and emission photons in the thin film formed by the hBN-material interfaces.^156,157^ A related behavior is observed in devices containing emitters in TMDs encapsulated in hBN dielectric layers, where the dielectric layers form a Fabry–Perot cavity that strongly influence the photon collection efficiency.^158^ These results evidence the importance of carefully designing the photonic environment of quantum emitters in vdW materials for effective technological implementations.

**Figure 4:**
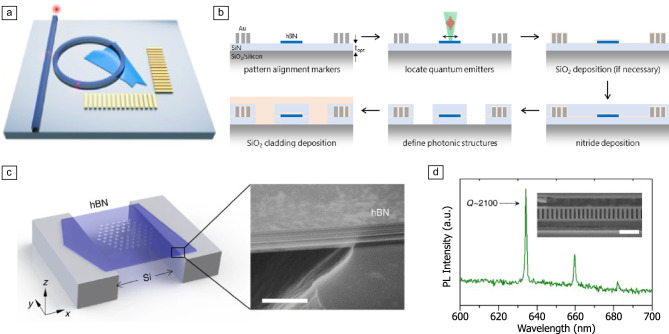
Photonic devices containing spin–photon interfaces (SPIs) in transition-metal dichalcogenides (TMDs) and hBN. (a) Schematic representation of a SiN resonator containing vdW materials, specifically hBN multilayers and TMD monolayers, embedded within the photonic structure, leading to enhanced coupling efficiency. This approach relies on overgrowth SiN around the active vdW material, as shown schematically in (b).^163^ (c) Schematic representation and scanning electron microscopy of a suspended multilayer hBN that can be subsequently patterned to create photonic structures.^164^ Scale bar = 500 nm. (d) Photoluminescence (PL) spectrum of an hBN monolithic photonic crystal cavity, shown in the inset, with quality factor approaching $\sim$2100.^164^ Scale bar = $1~{\upmu }$m.

Open, tunable microcavities containing TMDs and hBN with SPEs have been demonstrated to reduce excited-state lifetime, increase emitter brightness, and reduce inhomogeneous spectral broadening of the optical emission feature through the Purcell effect,^159–161^ albeit in a geometry that does not favor direct on-chip integration. In contrast, high-n photonic device architectures, including photonic resonators and waveguides based on established material platforms such as SiN enable direct integration of these systems with commercially available photonic device architectures.^54,162^ In this direction, recent approaches involve embedding TMD MLs with emitters within the photonic structure through overgrowth of SiN material^163^ (**Figure** **4**a–b). This allows for deterministic placement of quantum emitters within the photonic structure and enhances coupling efficiency up to $46\%$, a tenfold improvement on previous approaches based on transfer of vdW materials containing quantum emitters over the photonic devices.^54^

One path toward increasing the coupling efficiency of light coupling to SPIs in vdW materials is developing monolithic devices (e.g., photonic cavities,^164^ waveguides,^165^ and metasurfaces)^166^ where the vdW materials themselves act as high-n dielectric (Figure 4c–d). Both TMDs and hBN are highly suitable for the development of photonic structures,^104,164,166–168^ owing to their high refractive index,^169^ strong birrefringence, and compatibility with various nanofabrication techniques.^170^ However, these photonic devices typically require materials with few-nanometer thickness (i.e., several layers, see Figure 4c) in order to provide effective light coupling. As SPIs in TMDs are only stable at the ML or few-layer limit, this has so far hindered the development of monlithic devices integrating SPIs to photonic structures based on TMDs.^170^ For this reason, integration of SPIs in TMDs with photonic structures has so far relied on combining MLs of TMDs with widely established photonic materials such as SiN,^54,163^ LiNbO$_3$,^130^ GaP, and SiO$_2$.^171^

In contrast, hBN provides specific opportunities in this context: defect-based optically active spins in hBN are stable in multilayer and polycrystalline hBN structures that are typically necessary for efficient high-n photonic structures.^108^ These features have enabled the development of monolithic devices where quantum emitters are embedded within photonic waveguides and Bragg cavities with high-quality factors (Figure 4d),^104,164,165^ maximizing light–matter coupling efficiency and resulting in a $\sim$fivefold improvement of the photon collection rate from the quantum emitters in saturated conditions. Usual techniques used for spectral tuning of resonances of optical cavities, such as mass-shifts due to adsorption and desorption of molecules, have proven successful also for these hBN-based photonic structures,^104,164^ providing a pathway for spectrally stabilizing and controlling the optical emission spectrum of defects in hBN through the Purcell effect. Additionally, metasurfaces based on hBN have proven effective in providing photoluminescence-intensity enhancement and spectral narrowing of the optical features of spin-active defects in hBN,^166^ as well as an effective mechanism for manipulating the polarization and phase degrees of freedom of the emitted light from quantum emitters in the material.^172^

### Strain and spintronic devices

Although less explored in the literature, strain and spintronic devices hosting SPIs may also be important functional quantum technological components.

Due to the material dimensionality, vdW material platforms are highly susceptible to applied strain. Static strain devices where strain profiles are lithographically designed have been mentioned extensively as a means to obtain deterministic placement of SPIs in TMDs and hBN.^11,34,53,54^ Beyond these, active devices where strain can be reversibly applied to the vdW material host of SPIs provide a mechanism for spectral control of the optical transition of SPIs in both TMDs^173^ and hBN.^174^ In addition, devices with controlled strain have successfully enabled turning emitters on/off,^111,175^ providing a path to suppress background photon emission that degrades single-photon purity of SPIs in these materials. Mechanisms to reversibly control the strain applied to the vdW material platform include depositing the material onto flexible polymeric substrates^111,174^ or piezoactive substrates.^173,176,177^ Further, the possibility of processing vdW materials into few-atoms-thick membranes^178–180^ may be a facilitator for integrating SPIs to nanomechanical oscillators, enabling interconnects between photonic, spin, and mechanical degrees of freedom.^16^

Finally, spintronic devices encompass a varied class of device architectures where SPI-host materials are integrated with magnetically active (ferro, ferri, and antiferromagnetic) material platforms to provide spin-based added functionality. One material of interest for the development of such devices is yttrium iron garnet (YIG), a ferrimagnetic material characterized by ultralow magnetic damping responsible for efficient transport of magnons, GHz-to-THz magnetic excitations. Initial results based on devices incorporating YIG and diamond membranes hosting single NVs demonstrate that magnons can drive long-range NV-spin transitions in the GHz regime^14^ with two orders of magnitude reduction in the microwave power requirements.^181^ Related devices integrating YIG and optically active spins in hBN have been implemented,^182^ albeit only at the defect-ensemble level. Single-spin-magnon interactions for hBN-based SPIs have not yet been demonstrated. Alternatively, spintronic devices may incorporate vdW magnetic materials and SPI-hosts in order to explore proximity effects as a mechanism to imprint spin-polarization onto optically active single-photon emitters. Although proximity effects have been explored for modifying spin properties of charge carriers in vdW materials,^183–186^ their application toward tailoring localized-emitter properties has only recently been explored. Important demonstrations have been so far based on WSe$_2$ emitters, and include enhancement of the g-factor,^187^ spin initialization of localized emitters through spin-selective charge transfer to the ferromagnetic material,^188^ and chiral light generation due to proximity effects arising from coupling to a strained antiferromagnetic.^189,190^ Combined with the fact that the magnetization of vdW materials may be manipulated externally in spintronic devices, these results open the path for exploring spintronic device architectures capable of manipulating the quantum state of photons emitted by SPIs in vdW materials through the magnetization degree of freedom.^9^

## Pespectives and outstanding challenges

While well-established platforms such as epitaxially grown semiconductor quantum dots and defects in bulk diamond (e.g., the NV center) have paved the way for solid-state quantum technologies, optically active spins in 2D materials present a distinct set of advantages and challenges. The vdWs nature of these materials has significantly accelerated the exploration of device integration possibilities, enabling facile stacking and heterostructure formation that is difficult to achieve with lattice-matched bulk counterparts. This inherent versatility has enabled rapid prototyping of novel device architectures and exploration of proximity effects. However, despite this rapid progress, fundamental questions persist regarding the precise microscopic structure of many observed spin–photon interfaces, the comprehensive spectroscopic characterization required to fully understand and utilize their behavior, and the stringent requirements for creating stable and reproducible defects with desired quantum properties. The remarkable progress in understanding and manipulating optically active spins in 2D materials and devices opens exciting avenues for future quantum technologies, but addressing these critical challenges is essential to fully realize their potential.

A persistent challenge lies in the precise identification of the microscopic configurations responsible for optically addressable spins, particularly in materials such as hBN and TMDs. As discussed, the apparent discrepancy in creation strategies for similar quantum emitters in TMDs^61,62^ and the wide variety of spin defects emerging in hBN with similar optical properties, but distinct spin properties^83,90–92,109–112^ underscore this complexity. This difficulty hinders the reproducibility of experimental demonstrations and the design of deterministic fabrication approaches for specific defects. Overcoming this requires extended and integrated spectroscopy techniques capable of pushing the boundaries of spatial and spectral resolution—such as STM-PL,^33^ cathodoluminescence,^191^ integrated x-ray and PL studies,^192^ and electron spin resonance techniques with atomic resolution^193^—coupled with the development of advanced theoretical methods.^87^ These theoretical methods must be capable of encompassing the peculiarities of vdW materials, such as interlayer bonding, out-of-plane deformations,^107,127,128^ and complex charge-transfer dynamics within defect pairs.^118–120^ Innovative approaches combining meticulous material preparation, experimental characterization, and robust theoretical modeling, such as those applied to identify blue emitters in hBN,^98^ will be crucial for unraveling the atomic and electronic structure of these emitters. Eventually, opportunities may arise from the variety of carbon-related defects that seem to be stable in hBN, with further flexibility arising from defect-pair configurations. Understanding the behavior and formation pathways of these systems could lead to the design of defect clusters with large tunability for the internal photophysical rates that give rise to optical spin initialization.^123^

Additionally, the quality and scalability of SPIs are fundamentally tied to the underlying material growth techniques. Achieving high-quality materials with low residual doping is essential to minimize spectral wandering, blinking, and other detrimental charge-noise effects that impact optical linewidth and spin coherence.^39,41,56,57,141,142^ Simultaneously, the applicability of these platforms in quantum technologies necessitates the development of large-area growth techniques, such as chemical vapor deposition and pulsed laser deposition,^122^ capable of producing uniform and high-quality vdW materials hosting SPIs. Of the device architectures presented in this article, some will require the development of complex vdW-on-vdW heterostructures, which will only be scalable through controlled growth techniques. For example, integrating ML-TMD SPIs into monolithic photonic structures may be possible through the development of advanced heterostructures with alternating ML TMD and hBN layers to optimize both quantum emission and photonic functionality. This approach, however, demands sophisticated techniques for the controlled growth and stacking of such complex hBN/TMD/hBN heterostructures with precise control over interfaces and layer properties.

A final, overarching challenge is the development of multifunctional quantum devices that seamlessly integrate the various platforms and device architectures described in this article. This involves combining electronic devices for charge and spin control, photonic devices for enhanced light–matter coupling and photon collection, and spintronic/mechanical devices for magnetic field sensing, spin-magnon coupling, or coherent interfacing with other quantum systems. The ultimate goal is to create integrated systems where different degrees of freedom (electronic, photonic, spin, mechanical) can be manipulated and controlled. This requires addressing compatibility issues between diverse materials, developing advanced nanofabrication techniques for complex device geometries, and innovating strategies for efficient signal transduction between different device components. The progress toward robust and scalable quantum technologies will depend critically on our discovery of ways to engineer these multifunctional platforms by design.
